# Improving in-patient neonatal data quality as a pre-requisite for monitoring and improving quality of care at scale: A multisite retrospective cohort study in Kenya

**DOI:** 10.1371/journal.pgph.0000673

**Published:** 2022-10-20

**Authors:** Timothy Tuti, Jalemba Aluvaala, Daisy Chelangat, George Mbevi, John Wainaina, Livingstone Mumelo, Kefa Wairoto, Dolphine Mochache, Grace Irimu, Michuki Maina, The Clinical Information Network Group, Mike English

**Affiliations:** 1 KEMRI-Wellcome Trust Research Programme, Nairobi, Kenya; 2 Department of Paediatrics and Child Health, University of Nairobi, Nairobi, Kenya; 3 Nuffield Department of Medicine, University of Oxford, Oxford, United Kingdom; University of Embu, KENYA

## Abstract

The objectives of this study were to (1)explore the quality of clinical data generated from hospitals providing in-patient neonatal care participating in a clinical information network (CIN) and whether data improved over time, and if data are adequate, (2)characterise accuracy of prescribing for basic treatments provided to neonatal in-patients over time. This was a retrospective cohort study involving neonates ≤28 days admitted between January 2018 and December 2021 in 20 government hospitals with an interquartile range of annual neonatal inpatient admissions between 550 and 1640 in Kenya. These hospitals participated in routine audit and feedback processes on quality of documentation and care over the study period. The study’s outcomes were the number of patients as a proportion of all eligible patients over time with (1)complete domain-specific documentation scores, and (2)accurate domain-specific treatment prescription scores at admission, reported as incidence rate ratios. 80,060 neonatal admissions were eligible for inclusion. Upon joining CIN, documentation scores in the *monitoring*, *other physical examination and bedside testing*, *discharge information*, and *maternal history* domains demonstrated a statistically significant month-to-month relative improvement in number of patients with complete documentation of 7.6%, 2.9%, 2.4%, and 2.0% respectively. There was also statistically significant month-to-month improvement in prescribing accuracy after joining the CIN of 2.8% and 1.4% for feeds and fluids but not for Antibiotic prescriptions. Findings suggest that much of the variation observed is due to hospital-level factors. It is possible to introduce tools that capture important clinical data at least 80% of the time in routine African hospital settings but analyses of such data will need to account for missingness using appropriate statistical techniques. These data allow exploration of trends in performance and could support better impact evaluation, exploration of links between health system inputs and outcomes and scrutiny of variation in quality and outcomes of hospital care.

## Introduction

Neonatal (new-born children aged ≤ 28 days) deaths account for 47% of all under-five deaths with 37% of these deaths occurring in Sub-Saharan African (SSA) countries [1]. These deaths are largely attributable to preterm birth, sepsis and intrapartum complications [2] and hospital admissions with these conditions are still associated with high morbidity in Low- and Middle-Income Countries (LIMCs) like Kenya [3]. Essential interventions such as newborn resuscitation, Kangaroo Mother Care (KMC), early recognition and treatment of neonatal infections, and Continuous Positive Airway Pressure (CPAP) therapy have been identified as major interventions to reduce neonatal deaths in hospitals [4, 5]. However, available evidence suggests that adherence to recommended care giving practices in LMICs is poor [6–8] while poorly functioning information systems mean limited data of questionable quality on the delivery of such interventions in routine hospital settings in LMIC is available [9, 10]. This poor data quality precludes effective monitoring of the routine quality of care provided (where quality of care is defined as the adherence to the recommended clinical guidelines in provision of care) and patient outcomes at scale, and, limits the ability to track effective delivery of essential neonatal interventions.

Availability of high-quality timely, accessible, and easy to use data from routine clinical settings could improve monitoring of intervention adoption and quality of hospital care at scale, and ultimately might help improve clinical outcomes [9–12]. An integrated approach providing a mechanism to promote continued improvement of clinical information, implementation of effective practices and technologies, and locally relevant research can comprise a ‘learning health system’, which are posited to be influential in producing the positive change required [13–16].

The objectives of this study were to determine: (1) if the quality of documentation that is the source of routine data improves over time so that good quality data can be generated from hospitals’ newborn units invited to participate in a low-cost learning health system, (2) if basic recommended treatments or interventions are being correctly provided to neonatal in-patients (if the quality of clinical data permits this) and so explore the potential for tracking intervention adoption and ultimately their effects in LMIC.

## Methods

### Ethics and reporting

The reporting of this observational study follows the Strengthening of reporting of observational studies in epidemiology (STROBE) statement [17]. The Scientific and Ethics Review Unit of the Kenya Medical Research Institute (KEMRI) approved the collection of the de-identified data that provides the basis for this study as part of the Clinical Information Network (CIN) for newborns (CIN-N). Individual consent for access to de-identified patient data was not required.

### Ethics approval and consent to participate

Ethical approval was provided by the KEMRI Scientific and Ethical Review Committee (SERU 3459). Individual patient consent for the de-identified clinical data was judged to not be required, but consent from participating hospitals was sought.

### Study design and setting

This study is situated within the CIN-N. The CIN-N is a collaborative learning health system network between KEMRI-Wellcome Trust Research Programme, the Ministry of Health, Kenya Paediatric Association, and 21 partner hospitals [9, 18]. The hospitals in CIN-N are first referral-level, geographically dispersed hospitals with an interquartile range of annual NBU inpatient admissions between 550 and 1640. A paediatric network was established in 2013/2014 to improve care given to inpatient children [13]. After co-development work with a single large NBU, multiple hospitals’ neonatal units joined to extend the original paediatric network and create the CIN-N in 2017/2018. In these hospitals most admission care and prescribing is done by medical officer interns who rotate through departments regularly resulting in almost complete changes in those responsible for NBU admissions every three months [19]. In-depth description of the development of CIN-N and its activities are detailed elsewhere [3, 9, 13–16, 18]. For the purposes of this study, NBU data is omitted from analyses from one hospital because it was developing and using information tools with the CIN-N team for four years before any additional hospitals joined the CIN-N; thus, only data from 20 hospitals is analysed.

This was a retrospective cohort study involving NBUs in the CIN-N hospitals. The CIN-N hospitals receive three-monthly clinical audit and feedback reports on the quality of care including for example, summaries of key issues for documentation and treatment prescription errors once they join [18]. Shorter feedback reports on data quality, and morbidity and mortality reports are disseminated monthly via email to clinicians, nurses in-charge and other hospital administration staff. Neonatal team leaders (neonatologists, paediatricians, and nurses) met face to face once or twice annually until 2020 (before the COVID-19 pandemic) to discuss these reports and how to improve clinical care. Finally, those that received no feedback were neither included in written reports nor discussed in meetings. During the COVID-19 pandemic only short, online network meetings were conducted that focused mostly on disseminating information of relevance to the pandemic and efforts to improve local neonatal audit and nursing practices.

### Study size and participants

All hospitals have a specific newborn unit (NBU) and neonates aged ≤28 days admitted between January 2018 and December 2021 to the NBU of 20 CIN-N hospitals were eligible for inclusion. Neonates excluded were those whose admission or discharge dates were missing or improbable (e.g. discharge date is earlier than admission date), and those whose admissions fell within prolonged health worker strikes that resulted in major disruption to health care delivery (i.e. December 2020 –January 2021) [20].

### Data sources and management

Methods of collection and cleaning of data in the CIN-N are reported in detail elsewhere [21]. Clinical data for neonatal admissions to the hospitals within the CIN-N are captured through Neonatal Admission Record (NAR) forms and other forms and charts that are part of the hospital’s medical record. The NAR and associated patient charts prompt the clinician with a checklist of fields covering nine documentation domains that include demographics, admission information, discharge information, maternal history, presenting complaints, cardinal signs on examination, other physical examinations, nursing monitoring and supportive care [18]. Other charts that are also used are a comprehensive newborn monitoring chart (collects data on vital signs, feeds and fluids prescribed) which were developed and introduced between March and June 2019 [22], transfer forms (containing key data when a baby is transferred internally from maternity unit to NBU), treatment sheets, discharge summaries and death notification reports in case death occurs.

The clinical signs included in the NAR are based on recommendations in guidelines from the national Ministry of Health and the World Health Organisation (WHO) [23]. NAR forms were originally developed as part of the Emergency Treatment and Triage plus admission (ETAT+) approach which includes skill training in essential inpatient newborn care [24]. In earlier work they were associated with improved documentation of key patient characteristics during admission [18]. NAR are not provided to hospitals in CIN-N and so their adoption is at the discretion of hospital teams and supported by hospitals’ own resources, although CIN-N hospitals are encouraged to use them.

Each hospital has a clerk who extracts data from the NAR forms into a Research Electronic Data Capture (REDCap) database [25]. Two sets of data are captured: minimum and full datasets. The minimal dataset–which is unsuitable for this study’s analyses—is collected for (1) admissions during major holidays when the data clerk is on leave, and (2) on a random selection of records in hospitals where the workload is very high. The minimal dataset includes biodata and patient outcomes at discharge and is collected on all neonatal admissions in all CIN-N hospitals for reporting to the national Health Information System. The full dataset contains comprehensive data on admission details, patient history, clinical investigations, treatment and discharge information including diagnoses and outcome [9]. The data collected is subjected to routine quality assurance checks, with this process explained in detailed reports published elsewhere [9].

### Quantitative variables

#### Creation of documentation scores

The outcome for objective 1 of this study was based on use of individual patient documentation scores compiled from the signs, symptoms, treatments, and outcomes data (Table 1). These scores were developed for each of the eight NAR indicator documentation domains then used to determine trends in the completeness of documentation in the hospitals involved. Domains had different numbers of component data items (Table 1) considered key for characterising NBU populations and assessing core aspects of technical quality of care neonates receive [26]. Domain-specific composite scores for each patient were developed by arithmetic aggregation of all items with valid (non-missing) data in that domain (score = 1 if valid data, = 0 for missing data).

**Table 1:** Variables used in the documentation and coverage scores.

Documentation score			
Domain of care	Variables	Denominator	Maximum score^1^
**Demographics**	1. Sex 2. Age in days 3. Birth weight 4. Gestational age	All admissions	4
**Admission information**	1. Use of the Neonatal Admissions Record (NAR) 2. Date of birth 3. Date of admission 4. Admission weight 5. Mode of delivery 6. APGAR^2^ score at 5 minutes 7. Admission diagnosis	All admissions	7
**Discharge information**	1. Date of discharge/death 2. Outcome (dead/alive) 3. Discharge diagnosis 4. Discharge weight	All admissions/ those who died	4
**Maternal history**	1. Age 2. Parity 3. HIV status 4. VDRL status^3^	All admissions	4
**Presenting complaints (Newborn)**	1. Fever 2. Convulsions 3. Difficulty breathing 4. Difficulty feeding 5. Apnoea	All admissions	5
**Cardinal signs on examination**	1. Grunting 2. Central Cyanosis 3. Bulging fontanelle 4. Floppy (inability to suck reduced movements / activity)	All admissions	4
**Other physical examination and bedside testing**	1. Pallor/Anaemia 2. Bilateral air entry on chest examination 3. Chest indrawing 4. Capillary refill time 5. Signs of umbilical infection 6. Irritability 7. Jaundice 8. Glucose test	All admissions	8
**Monitoring (Vital signs)^4^**	Nursing chart: 1. Temperature 2. Pulse rate 3. Respiratory rate 4. Pulse oximetry or central cyanosis	All admissions	4
**Coverage score^5^**	**Variables**	**Denominator (eligible neonates)**	**Maximum score**
	1. Repeated weight monitoring	Birth weight less <2.5 kg and length of stay >6 days	N/A
	2. CPAP^6^	A clinical diagnosis of respiratory distress syndrome at admission or discharge	N/A
	3. KMC^7^	Birth weight <2kg discharged alive	N/A

Note

^1^ These values represent the maximum number of indicators per domain

^2^APGAR: **A**ppearance (skin colour); **P**ulse (heart rate); **G**rimace response (reflexes); **A**ctivity (muscle tone); **R**espiration (breathing rate and effort)

^3^VRDL: **V**enereal **D**isease **R**esearch **L**aboratory

^4^Monitoring (Vital signs) documentation domain represents ONLY the first admission set of vital signs

^5^Percentage of eligible neonates who received the intervention.

^6^CPAP: **C**ontinuous **P**ositive **A**irway **P**ressure

^7^KMC: **K**angaroo **M**other **C**are

#### Creation of treatment correctness scores and intervention tracking

An additional three indicator domains were created to reflect the accuracy of basic treatment prescriptions (antibiotics, fluids, and feeds) for relevant sub-populations of neonates receiving these treatments and based on the dosage or volume recommendations in the national guidelines [27] (Table 2). Each eligible patient in each of the treatment domains in the analysis could either have correct or incorrect prescription: If the treatment was correctly prescribed, then it contributed a score of one to the domain-specific score; if treatment information was missing or treatment was incorrectly prescribed, then it contributed a score of zero to the domain-specific score. Finally, descriptive analyses was done to assess how well the data could support tracking of intervention adoption over time by evaluating whether neonates eligible for weight monitoring, CPAP, and KMC received these essential services. The adoption of weight monitoring, CPAP and KMC was summarised by coverage scores calculated as the percentage of neonates potentially eligible who were recorded as receiving these interventions/monitoring.

**Table 2:** Threshold for prescribing antibiotics, fluids, and feeds.

Domain (Dose determination) ^1^		Correct treatment	Underdose	Overdose	Documentation Variables	Denominator	Maximum score^2^
**Antibiotic treatment** (Dose prescribed/birth weight in kilograms)	Penicillin	1. 40000 ui/kg to 60000ui/ kg for 12-hourly for age <7 days or, 2. 40000 ui/kg to 60000ui/ kg for 8-hourly for age 7–28 days	<40000 ui/kg^3^	>60000 ui/kg^4^	1. Dose 2. Frequency 3. Route 4. Age 5. Birth weight	Patients prescribed with penicillin	5
	Gentamicin	1. ≥2.4 mg/kg to ≤3.6 mg/kg for birth weight <2 kg and age <7 days, or 2. ≥4 mg/kg to ≤6 mg/kg for birth weight ≥ 2kg and age <7 days, or 3. ≥6 mg/kg to ≤9 mg/kg for age 7–28 days	1. <2.4 mg/kg for birth weight < 2kg and age <7 days, or 2. <4.0 mg/kg for birth weight ≥ 2kg and age <7 days 3. <6 mg/kg for age 7–28 days	1. >3.6 mg/kg for birth weight <2 kg and age <7 days or, 2. >6 mg/kg for birth weight ≥2 kg and age <7 days 3. >9 mg/kg for age 7–28 days	1. Dose 2. Frequency 3. Route 4. Age 5. Birth weight	Patients prescribed with gentamicin	5
**Fluids^5^** (Intravenous fluids prescribed / birth weight in kilograms)		1. ≥64 ml/kg/day to ≤96 ml/kg/day for birth weight <1.5kg or, 2. ≥48 ml/kg/day to ≤72 ml/kg/day for birth weight ≥1.5 kg	1.<64 ml/kg/day for birth weight <1.5 kg or, 2. <48 ml/kg/day for birth weight ≥1.5 kg	1. >96 ml/kg/day for birth weight <1.5 kg or, 2. >72 ml/kg/day for birth weight ≥1.5 kg	1. Volume 2. Duration 3. Age 4. Birthweight	Patients with fluid prescription	4
**Feeds^5^** ((volume of feeds prescribed in milligrams / birth weight in kilograms) number of times administered)		1. ≥64 ml/kg/day to ≤96 ml/kg/day for birth weight <1.5kg or, 2. ≥48 ml/kg/day to ≤72 ml/kg/day for birth weight ≥1.5 kg	1. <64 ml/kg/day for birth weight <1.5kg or, 2. <48 ml/kg/day for birth weight ≥1.5 kg	1. >96 ml/kg/day for birth weight <1.5 kg or, 2. >72 ml/kg/day for birth weight ≥1.5 kg.	1. Type 2. Volume 3. Frequency 4. Age 5. Birthweight 6. Route	Patients with enteral feeds prescribed	6

Note

ml–milliliters; mg–milligrams; ui–international units; kg–kilograms

^1^Allows for ±20% error margin in prescription dose

^2^ These values represent the maximum number of indicators per domain

^3^ Includes dose given fewer times than 12-hourly for age < 7 days or 8-hourly for age 7–28 days respectively.

^4^ Includes dose given more times than 12-hourly for age < 7 days or 8-hourly for age 7–28 days respectively.

^5^ For prescriptions given to neonates in day one of life. Feeds prescribed covers only enteral feeds and does not include any trophic feeds given. Intravenous fluids prescribed covers only 10% Dextrose (D10) prescriptions.

### Statistical methods

#### Descriptive analyses

Documentation performance of each hospital over time was summarised monthly using trend plots as a percentage representing (1) the average score of individual patient domain-specific score as a proportion of the maximum domain score possible (i.e., Documentation score), and (2) the number of patients with maximum possible documentation score for each domain out of all patients with full data collection admitted to the NBU. Similarly, domain-specific treatment accuracy and treatment coverage was summarised monthly for each hospital using trend plots as a percentage representing the proportion of neonates eligible for essential treatments who received an accurate prescription or required intervention respectively.

These pooled scores are presented using scatter plots for each hospital each month over the period 2018 to 2021 (i.e., from the month of joining CIN-N). Locally Weighted Scatterplot Smoothing (LOWESS) line plots were used to visually represent the trend over time for each documentation, treatment accuracy, and treatment coverage domain. Descriptively, from the hospital-specific trend plots, hospitals whose performance at the month of joining CIN is below 40% were considered to have a low baseline while those with a performance above 75% were considered to have a high baseline.

#### Inferential statistics

To characterise clinical documentation completeness and adherence to recommended treatment prescribing guidelines over time while quantifying heterogeneity between CIN-N hospitals, generalised linear mixed effects models were fitted for each documentation and treatment accuracy domain. These mixed effects models (using log link) were fitted on two types of hospital-level count outcome variables computed monthly:

- Documentation domain completeness score per hospital (as the number of all patients with all domain-specific variables documented out of all patients admitted to CIN-N hospitals).
- Treatment domain accuracy score per hospital (as the number of all patients with accurate treatment prescription out of all those with the treatment prescribed).

For our approach to the mixed effects model fitting, the patients are nested in hospitals nested in time points. Time elapsed was captured as months since the hospitals joined the CIN-N and was treated as a continuous fixed effect. From previous studies within the CIN, the effect of time on adherence to recommended clinical practice was found to vary across hospitals [28]. Likelihood ratio tests (LRT) were used to determine the most suitable random effects model (hospital random intercepts vs hospital random intercepts with random slopes for time). The outcome variables for documentation and treatment domains were assumed to follow a negative binomial distribution (a generalisation of Poisson regression which loosens the restrictive assumption made by the Poisson model that the variance is equal to the mean i.e. equidispersion assumption) [29]. The sensitivity analyses sub-section of the methods section addresses how this assumption was tested. An offset term (i.e., the number or patients eligible per month per hospital) was included in each model to model the count outcome as a rate over time (e.g., change in the number of patients with accurate treatment prescribed), and the model effects are reported as incidence rate ratios (IRR). Intra-cluster correlation coefficients (ICC) were provided to indicate variation between hospitals in recommended documentation practices and adherence to treatment guidelines.

#### Missing data

Missing data was considered ‘informative’ as the analysis is based on documentation or no documentation. For the documentation score, missing variables were recorded as zero and therefore contributed a score of zero to the domain score per patient. For treatments, the absence of clear prescription information was logically considered to represent an inadequate prescription; for coverage, no record of use of the intervention was assumed to indicate no use.

#### Sensitivity analyses

Overdispersion of the outcome variables (which is when the conditional variance exceeds the conditional mean), a key negative binomial model assumption, was evaluated by a likelihood ratio test comparing the model(s) to their Poisson model equivalent, which holds the conditional mean and variance to be equal (i.e., Equidispersion). Also, a likelihood ratio test (LRT) was used to examine the most suitable random effect model (random intercepts at the hospital level versus random intercepts for the hospitals with random slopes for time) [30]. To ensure that the correlations between the repeated outcome measurements of each hospital which decrease with time lag (i.e. autocorrelation) were adequately reflected, LRT was used to examine if there was evidence to support including a term for an autocorrelation structure of order one [31], over using a mixed effects model without such a term.

Finally, exploration of whether there was evidence supporting the assumption that the conditional outcome of the models’ approximated a normal distribution using quantile residual quantile-quantile (QQ) plot for each fitted model was conducted, although this assumption is debatable for count data models [32].

## Results

### Descriptive findings

**Fig 1:**
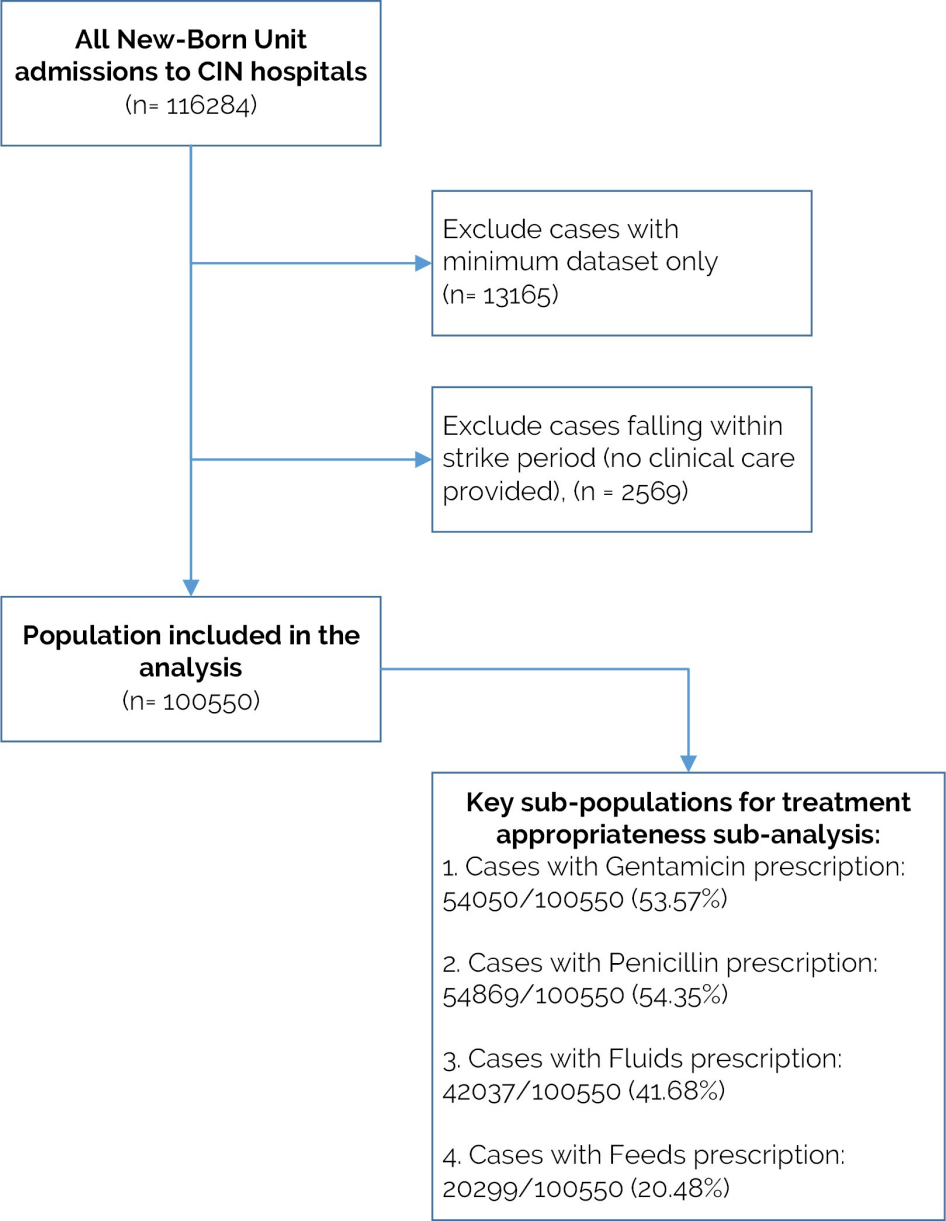
Flow-chart of the inclusion criteria. The overall population (n = 80060) is used for documentation score analysis. S1 Table in supporting information provides details of when CIN-N hospitals joined the network and patient records per hospital so far.

Fig 1 depicts the study population inclusion process. Out of the 84,960 NBU admissions to CIN-N hospitals, 80,060 (94.23%) were eligible for analysis. Most exclusions were because an admission was randomly sampled for minimum data collection (2934/84960) or fell in industrial action period (1966/84960). Among the patients admitted to CIN-N during the study period and selected for this study, 43953/80060 (54.9%) were male. Overall, the mortality rate across the 20 hospitals was 11314/80060 (14.13%). The median birth weight of CIN-N NBU admissions was 3 kgs (inter-quartile range (IQR): 2.0–3.395) and median length of stay was 4 days (1QR: 2–8). NBU admissions had a median of one admission diagnosis (IQR: 1–2). The leading NBU discharge diagnoses over time was low birth weight followed by birth asphyxia, respiratory distress syndrome, and then neonatal sepsis. Out of the 42998/80060 (53.71%) NBU admissions with Gentamicin prescription and 43889/80060 (54.82%) with Penicillin prescription, 1022/42998 (2.38%) and 1964/43889 (4.47%) were classified as incorrect because of incomplete prescribing data (e.g., any of missing age, birth weight, dosage, route, and frequency of administration variables) respectively. Out of the 35295/80060 (44.09%) NBU admissions with fluids prescription and 13643/80060 (17.04%) with feeds prescription, 2778/35295 (7.87%) and 3721/13643 (27.27%) were classified as incorrect because of incomplete prescribing data respectively. The proportion of records in which key items are not recorded is illustrated in Table 3.

**Table 3:** Proportion of records for admission observations included in this study in which key items are not recorded.

Indicator domain	Variable	Patients with variable not recorded (%)
**Demographics**	Sex	823/80060 (1.03%)
	Age in days	282/80060 (0.35%)
	Birth weight	1597/80060 (1.99%)
	Gestational age	11552/80060 (14.43%)
**Admission Information**	NAR used	4895/80060 (6.11%)
	Date of birth	0/80060 (0%)
	Date of admission	0/80060 (0%)
	Admission weight	8008/80060 (10%)
	Date of delivery	1105/80060 (1.38%)
	APGAR Score (5 minutes)	7728/80060 (9.65%)
	Admission diagnosis	548/80060 (0.68%)
**Discharge Information**	Date of discharge/death	0/80060 (0%)
	Outcome	112/80060 (0.14%)
	Discharge diagnosis	2220/80060 (2.77%)
	Discharge weight	20054/80060 (25.05%)
**Maternal History**	Age	9770/80060 (12.2%)
	Parity	12742/80060 (15.92%)
	Gravidity	69365/80060 (86.64%)
	HIV status	7806/80060 (9.75%)
	VDRL status	10896/80060 (13.61%)
**Presenting Complaints (Newborn)**	Fever	7637/80060 (9.54%)
	Convulsions	8456/80060 (10.56%)
	Difficulty breathing	7015/80060 (8.76%)
	Difficulty feeding	9078/80060 (11.34%)
	Apnoea	8813/80060 (11.01%)
**Cardinal signs on examination**	Grunting	7541/80060 (9.42%)
	Central cyanosis	4818/80060 (6.02%)
	Bulging fontanelle	7042/80060 (8.8%)
	Floppy	7541/80060 (9.42%)
**Other physical examination and bedside testing**	Pallor/Anaemia	5454/80060 (6.81%)
	Bilateral air entry	10599/80060 (13.24%)
	Indrawing	7374/80060 (9.21%)
	Capillary refill	18020/80060 (22.51%)
	Umbilical infection	8682/80060 (10.84%)
	Jaundice	5691/80060 (7.11%)
	Glucose ordered	6786/80060 (8.48%)
	Irritability	12238/80060 (15.29%)
Monitoring (Vital signs)^1^	Temperature	27190/80060 (33.96%)
	Pulse rate	28417/80060 (35.49%)
	Respiratory rate	30613/80060 (38.24%)
	Pulse Oximetry / Central Cyanosis	38196/80060 (47.71%)
**Coverage score indicators**	Kangaroo Mother Care (KMC)^2^	10208/18102 (56.39%)
	Continuous Positive Airway Pressure (CPAP)^3^	1818/22431 (8.1%)
	Weight monitoring^4^	1118/13832 (8.08%)

Note

^1^Monitoring (Vital signs) documentation domain represents ONLY the first admission set of vital signs

^2^Denominator is neonates with birth weight <2kg discharged alive

^3^Denominator is neonates with a clinical diagnosis of respiratory distress syndrome at admission or discharge

^4^Denominator is neonates with birth weight less <2.5 kg and length of stay >6 days

### Objective 1 findings: Quality of documentation of in-patient neonatal care provided over time

**Fig 2:**
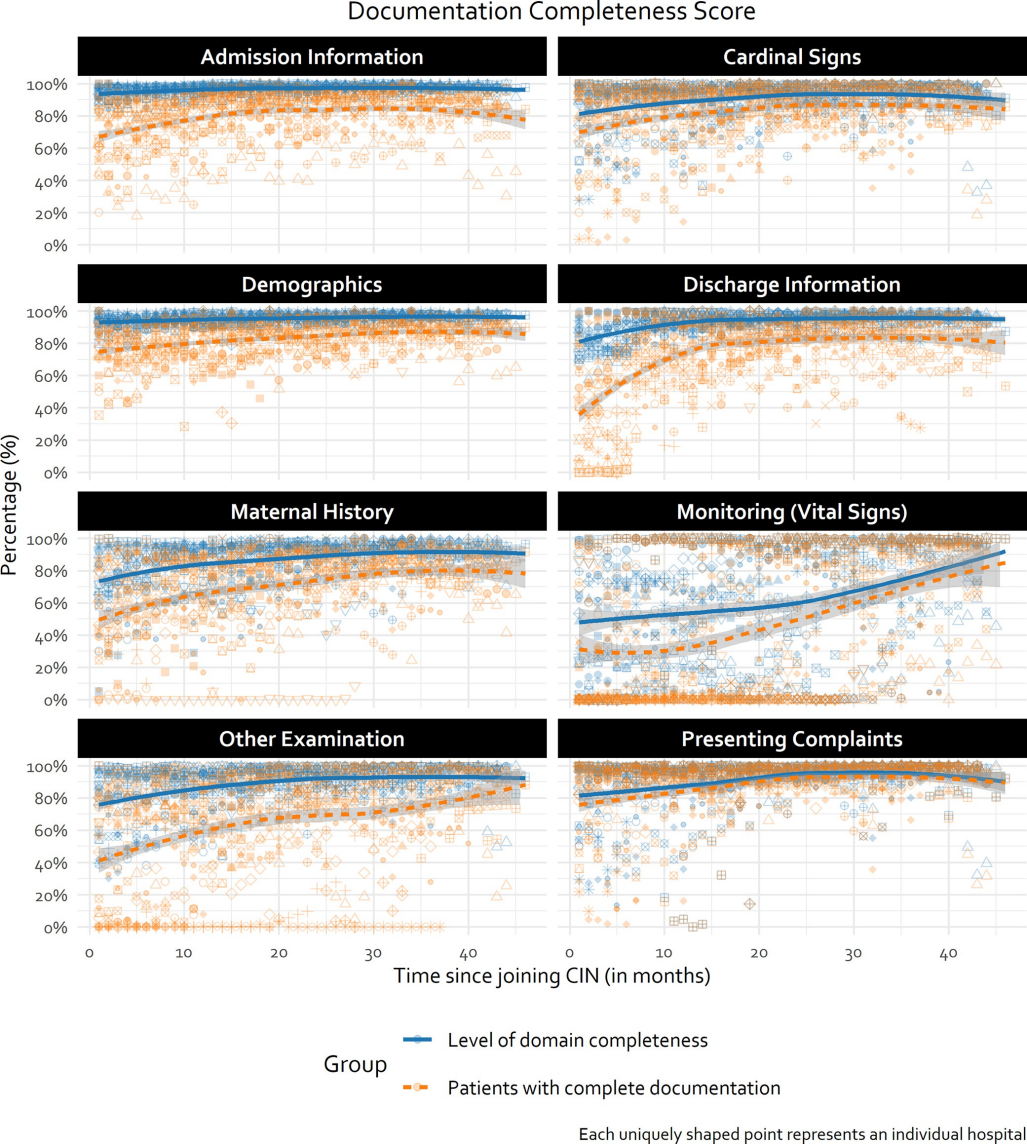
Domain-specific documentation trends over time. Domain completeness score summarised as an average of all individual patient domain-specific scores in each month. Trend line generated using LOWESS technique.

Examining trends with data from across hospitals it can be seen that at the time all (new) hospitals joined the CIN-N, documentation completeness for 5/8 documentation domains was already around 80% or better (Fig 2, Table 4). This is likely attributable to most of these sites already using the NAR linked to being already part of CIN-Paediatrics [9, 14, 16]. Specifically, for *admission information*, *discharge information* and *demographics* documentation domains, performance was consistently >95%, with a median of >80% of patients having full documentation at admission (Table 4, Fig 2). For this reason, further examination of hospital specific trends for these domains was not done.

**Table 4:** Domain documentation summary statistics pooled across all time periods (n = 80060 patients).

Domain of care	Median percentage (Inter-quartile range)	
	Level of completeness of domain documentation across time	Patients with complete domain documentation across time
Demographics	96.1% (93.34%-97.61%)	85.1% (75.47%-90.67%)
Admission information	97.7% (95.42%-98.59%)	85.4% (72.71%-91.11%)
Discharge information	94.8% (90.22%-97.64%)	81.6% (63.79%-91.39%)
Cardinal Signs	94.4% (88.07%-98.05%)	87.5% (76.16%-95.43%)
Maternal history	91.4% (81.23%-95.12%)	78% (58.56%-87.01%)
Presenting complaints (Newborn)	96.4% (90.7%-99.08%)	93.8% (85.23%-98.5%)
Other physical examination and bedside testing	92.7% (84.83%-97.28%)	74.9% (46.8%-90.14%)
Monitoring (Vital signs)	70.5% (27.13%-94.46%)	44.3% (0%-91.47%)

**Fig 3:**
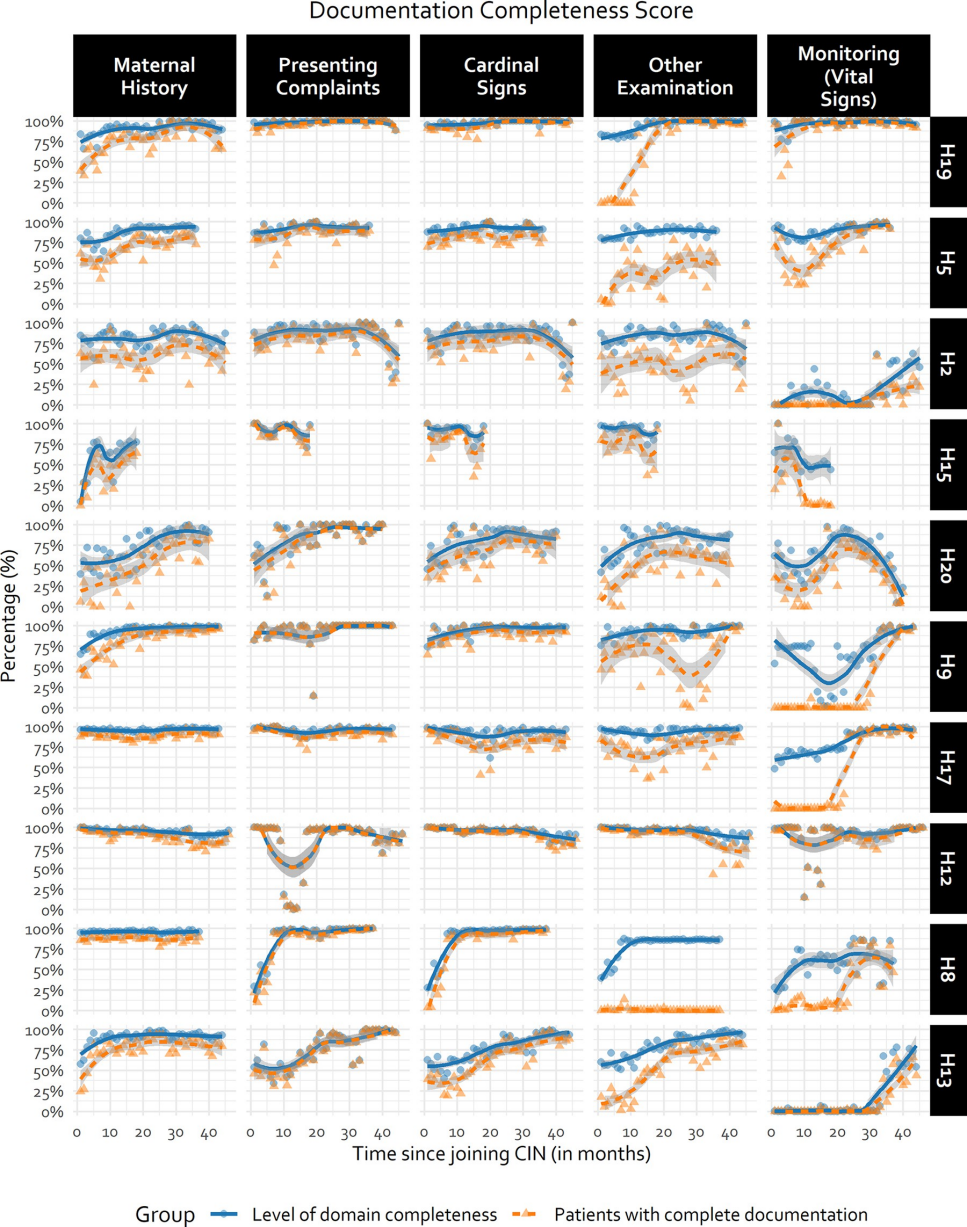
Illustration of hospital-specific documentation trends using a random selection of half the CIN-N hospitals. Hospital-specific trends for the remaining subset of hospitals can be found in S1 Fig of supporting information. Trend line generated using LOWESS technique. Fewer observations in some hospitals due to different CIN-N joining dates.

For other domains, performance started lower with a suggestion from all hospitals’ data of improvement over time, but also considerable between-hospital variability e.g., *maternal history*, *other bedside examination*, and *monitoring* (*vital signs*) (Fig 2). Fig 3 (and S1 Fig in supporting information) explored and demonstrated considerable between-hospital variability (e.g., *Other examination* domain). Plots display some examples of broad improvement (H13 and H20), some with static performance over time (e.g., H12) and some with rather erratic performance including occasional substantial declines (e.g., H2).

All documentation domains demonstrated month-to-month improvements in the number of patients with complete domain documentation–even if modest in size–which were statistically significant (Table 5); In descending order, *monitoring (vital signs)*, *other physical examination and bedside testing* (i.e. Other Signs), *discharge information*, and *maternal history* domains demonstrated a statistically significant month-to-month relative improvement in number of patients with complete documentation of 7.6%, 2.9%, 2.4%, and 2.0% respectively (Table 5).

**Table 5:** Incidence rates of complete domain documentation at admission over time.

Count models with random intercept for hospitals and autocorrelation term for time respectively^1^					
Domain	Term^2^	IRR	95% CI	P-Value	ICC (Correlation)^3^
Admission Information	(Intercept)	0.695	0.637–0.758	<0.001	0.6682 (0.988)
	Time	1.006	1.004–1.007	<0.001	
Cardinal signs on examination	(Intercept)	0.665	0.573–0.772	<0.001	0.66 (0.943)
	Time	1.007	1.002–1.012	0.009	
Demographics	(Intercept)	0.755	0.716–0.796	<0.001	0.6848 (0.976)
	Time	1.004	1.002–1.005	<0.001	
Discharge Information^4^	(Intercept)	0.344	0.258–0.458	<0.001	0.0239 (0.954)
	Time	1.024	1.014–1.034	<0.001	
Maternal History	(Intercept)	0.373	0.254–0.547	0.004	0.5341 (0.984)
	Time	1.020	1.01–1.03	0.022	
Monitoring (Vital signs)^4^	(Intercept)	0.028	0.01–0.078	<0.001	0.5121 (0.982)
	Time	1.076	1.046–1.107	0.006	
Other physical examination and bedside testing^4^	(Intercept)	0.206	0.11–0.388	<0.001	0.5064 (0.991)
	Time	1.029	1.016–1.043	<0.001	
Presenting Complaints	(Intercept)	0.711	0.618–0.819	<0.001	0.6752 (0.905)
	Time	1.008	1.002–1.013	0.006	

^*1*^ Time treated as continuous covariate representing consecutive months in CIN

^2^Intercept term provides baseline rate at month 1 of joining the CIN.

^3^Intra-Cluster Correlation (ICC), with autocorrelation parameter provided in the brackets. The choice to account for heterogeneity between hospitals while allowing for varying effects of time (i.e., a random slope) within each hospital was more suitable that just a hospitals random intercepts model; However, a hospitals random intercept model with an autocorrelation term for time outperformed a random slopes model equivalent (S2 Table in supporting information).

^4^Used negative binomial models in place of Poisson models given the equidispersion assumption violation (S2 Table in supporting information)

At the time of joining CIN-N, less than 50% of the patients admitted to the hospitals had complete documentation of *Discharge information*, *Monitoring (Vital signs)*, *Other physical examination and bedside testing*, and *Maternal history* domains, with a substantive amount of variance in the outcome explained by hospital factors as illustrated by the high ICCs (Table 5, Fig 3). Hospitals with higher baseline performance tended to demonstrate slower rates of improvement than hospitals with lower baseline performance as illustrated in Fig 3 (Table 5, H13 versus H17 in Fig 3).

### Objective 2 findings: Accuracy of essential neonatal intervention prescriptions over time

**Fig 4:**
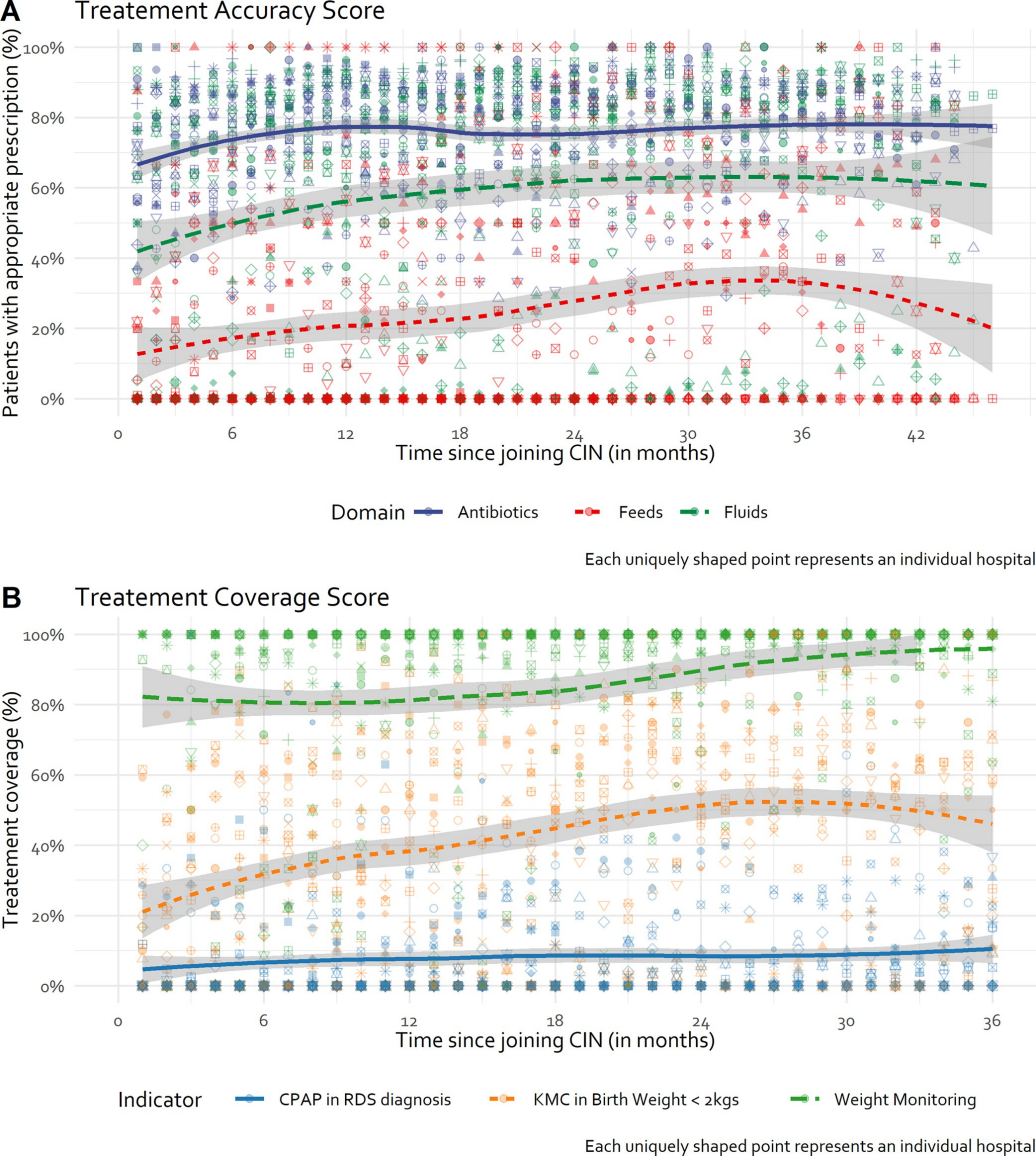
Overall trend in treatment accuracy and coverage. KMC: Kangaroo Mother Care; CPAP: Continuous Positive Airway Pressure; RDS: Respiratory Distress Syndrome. Trend line generated using LOWESS technique.

Given the good quality of prescribing data from CIN-N and the reasonable assumptions about the meaning of missing prescribing data, treatment prescribing accuracy was evaluated for the common antibiotics, feeds, and fluids in NBUs. Domain specific treatment accuracy scores revealed an increasing proportion of patients with accurate fluids and feeds prescriptions from approximately 40% to 60%, and 15% to 40% respectively, although feeds prescribing accuracy then regresses to 25% (Fig 4).

Antibiotic prescription shows a modest improvement in accuracy from around 65% to 80% within the first 12 months, after which it then fluctuated around 80% over time across all CIN-N admissions. Treatment coverage levels for KMC demonstrated an increase over time from 20% to 40% in neonates with birth weight <2kg (Fig 4). There was a small increase in CPAP coverage levels over time in neonates with a clinical diagnosis of respiratory distress syndrome (RDS) from 4% to 10%. Repeated weight monitoring for sick neonates improved from 80% to approximately 95% over time (Fig 4). There is evidence of moderate to high hospital variability in both treatment accuracy and coverage scores in CIN-N hospitals (Fig 4).

**Fig 5:**
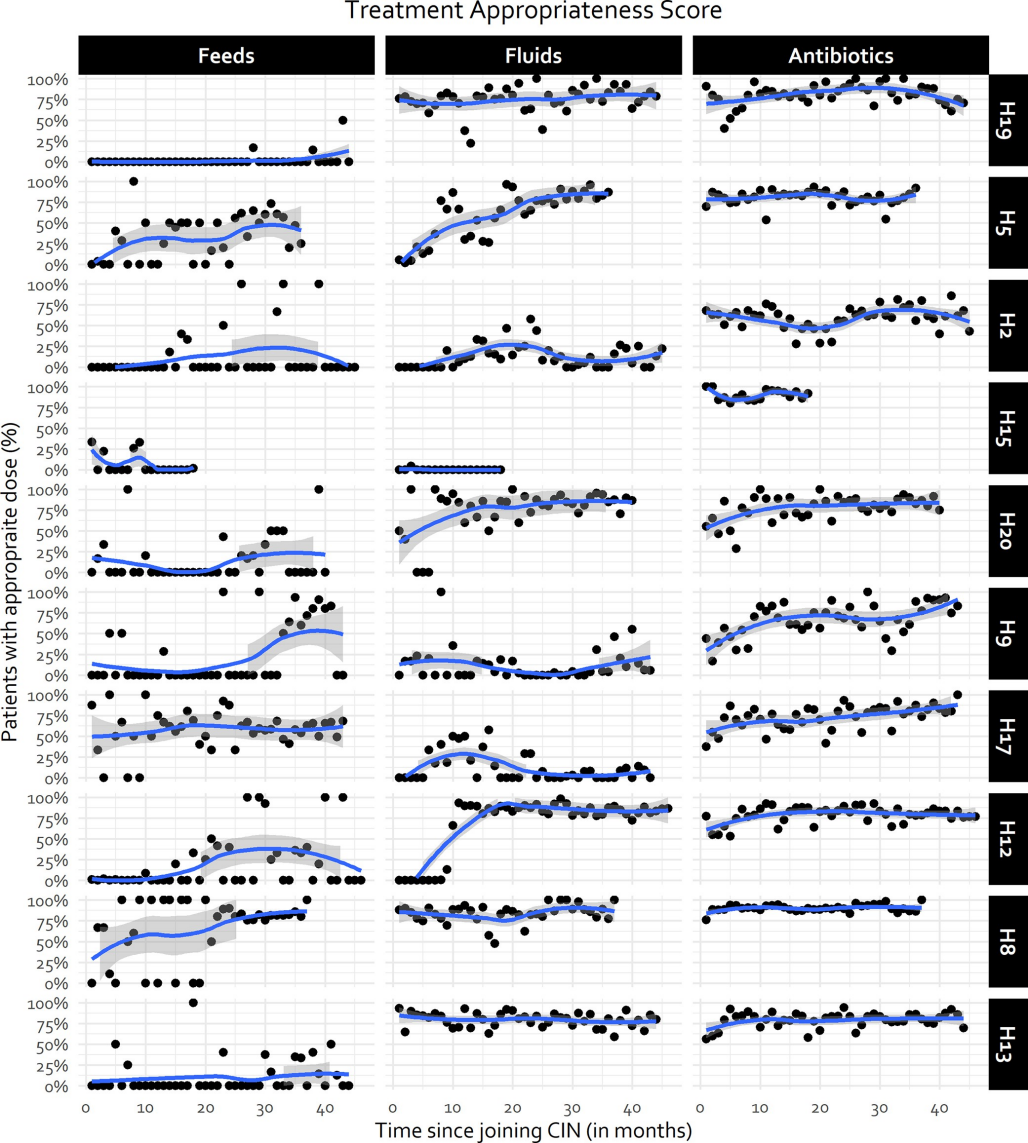
Hospital-specific treatment accuracy trends for half of randomly selected CIN-N hospitals. Hospital-specific trends for the remaining subset of hospitals can be found in S2 Fig in supporting information. Trend line generated using LOWESS technique. Fewer observations in some hospitals due to different CIN-N joining dates.

While antibiotic treatment accuracy seemed to have a ceiling effect of 80% in pooled hospital data, from hospital specific plots (Fig 5, S2 Fig in supporting information), some hospitals consistently attained accuracy levels > 80% (H8, H11, H19), an indication that improvement in other sites was possible. Hospital-specific trends suggested that fluids prescribing accuracy improved from a lower baseline for some hospitals (e.g., H5, H12, H20) but there is still a long way to go, with considerable between hospital variability evident over time (Fig 5, S2 Fig in supporting information). Similarly, feeds prescribing accuracy shows some improvement in some hospitals from a lower baseline (e.g., H5, H8) but in others performance is erratic (H2, H12), with most performing consistently poorly over time (e.g., H13, H19) (Fig 5, S2 Fig in supporting information).

On average, 73.5%, 10.8% and 22.8% of the patients in the CIN-N received the correct antibiotic, feeds, and fluids treatment at the time when hospitals joined the CIN-N (Table 6). There was a modest statistically significant month-to-month relative increase in correct inpatient treatment after joining the CIN of 2.8% and 1.4% for feeds and fluids prescribing accuracy. Antibiotic prescriptions showed no statistically significant month-to-month improvement after joining CIN-N (Table 6). The high ICC from the antibiotics and fluids mixed effects models suggests that much of the variation in the prescribing practices accuracy was associated with hospital-level factors.

**Table 6:** Incidence rates of correct treatment prescribing at admission over time.

Count model with random intercept for hospitals and autocorrelation term for time respectively^1^					
Treatment Domain	Term	IRR	95% CI	P-value	ICC (Correlation)^2^
Antibiotics	(Intercept)	0.735	0.688–0.786	<0.001	0.6771 (0.989)
	Time	1.002	1–1.003	0.053	
Feeds	(Intercept)	0.108	0.066–0.177	<0.001	0.0403 (0.984)
	Time	1.028	1.013–1.043	<0.001	
Fluids	(Intercept)	0.228	0.11–0.473	<0.001	0.4072 (0.995)
	Time	1.014	1.002–1.027	0.028	

^**1**^ Time treated as a continuous covariate representing consecutive months since joining the CIN; Months with no patients reporting assumed to have a MNAR mechanism and dropped from regression analysis.

^2^Intra-Cluster Correlation, with autocorrelation parameter provided in the brackets. The choice to account for heterogeneity between hospitals while allowing for varying effects of time (i.e., a random slope) within each hospital was more suitable that just a hospitals random intercepts model; However, a hospitals random intercept model with an autocorrelation term for time outperformed a random slopes model equivalent (S2 Table in **supporting information**).

### Sensitivity analyses findings

It was reasonable to use Poisson models (which assumes equidispersion) in all but four documentation domains (*Discharge information*, *Monitoring (Vital Signs)*, *Other physical examinations and bedside testing (i*.*e*., *Other Signs)* documentation domains) and the *Feeds* treatment accuracy domain which showed evidence suggestive of overdispersion (S2 Table, S3–S5 Figs in supporting information). Where the equidispersion assumption was violated, negative binomial models were used and informed any inference drawn.

## Discussion

### Summary of findings

This study aimed at determining if quality routine clinical data might be generated from CIN-N hospitals and if the quality of data improved over time. As the data quality was reasonable, they were then used to determine whether essential treatments or interventions are being correctly prescribed to newborns and to track intervention adoption. From the time hospitals joined the CIN-N, around 80% of newborns had complete documentation in 5/8 documentation domains (Table 4). This relatively good performance at baseline may be a consequence of participation in the paediatric CIN by most of these hospitals prior to formal extension of CIN to NBUs (i.e., CIN-N) with many paediatric practitioners previously exposed to use of the NAR, ETAT+ training and national neonatal guidelines [13]. All documentation domains demonstrated month-to-month statistically significant albeit modest improvements (between 0.6% and 7.6% per month) in the number of patients with complete domain documentation (Fig 4, Table 5). On average, 73.5%, 10.8% and 22.8% of the newborns with treatment orders in the CIN-N for first-line antibiotics, feeds, and fluids had correct prescriptions at the time when hospitals joined the CIN-N (Table 6). There is a modest statistically significant 2.8% and 1.4% month-to-month relative increase in accurate feeds and fluids prescription after joining the CIN-N resulting in an end line performance of around 40% and 60% respectively. Antibiotic prescribing showed no statistically significant month-to-month change.

Although sometimes modest the improvements observed were often sustained during the COVID-19 pandemic (perhaps with the exception of feed prescribing) that restricted network engagement activities to brief online meetings between April 2020 and December 2021. Improvements also occurred over a period of 4 years during which junior medical staff on NBUs changed every 3 months with frequent changes also in senior staff [19]. Across the entire period CIN-N sustained distribution of feedback reports and the magnitude of improvements observed are in keeping with findings from many audit and feedback interventions [33].

Coverage levels for KMC in neonates with birth weight <2kg and CPAP in neonates with clinically diagnosed respiratory distress syndrome (RDS) demonstrated an increase over time from 20% to 40% and 4%-10%; repeated weight monitoring for sick neonates with birth weight <2.5 kg and length of stay >6 days improved from 80% to approximately 95% over time (Fig 4). There was evidence of moderate to high hospital variability in documentation, treatment accuracy, and coverage scores in CIN-N hospitals; as shown previously hospitals with higher baseline performance evidence slower rates of improvement than hospitals with lower baseline performance in some cases perhaps linked to ceiling effects [34].

### Comparison to other findings

Previous studies in Kenya depict a health system that is struggling to collect quality data that is usable for decision making especially for neonatal care [18, 35]. The poor quality of neonatal clinical data has been widely reported in other African countries [36, 37], this undermines efforts to track the scale up of quality care [38, 39]. Implementation of a learning health system across hospitals utilising a common data platform to facilitate routine audit and feedback cycles have been shown to improve the documentation of patient data and its subsequent use in care improvement [9, 38, 40]. Employing findings, tools and practices from previous studies and progressively engaging more hospitals, this study demonstrated that data can be collected using a common data platform as part of a learning health system approach from a network of hospitals’ NBUs; this study further shows that these data can be useful for identifying potential gaps in care (e.g., treatment accuracy) with an aim of improving the quality of care provided in facilities and tracking outcomes at scale [13–16, 18, 41]. To our knowledge this is the largest reported long-term neonatal learning health system platform in SSA, serving as an exemplar actionable health information system in line with WHO standards [12, 13, 15, 16].

Findings from scoping reviews suggest that having better data can help improve quality of care if coupled with development of local leadership, training, and use of local improvement strategies such as mortality audits or quality improvement cycles; This can help reduce inpatient neonatal mortality in low-income country hospitals [42–45]. However, the complex intervention strategies required to tackle multiple quality and safety concerns in hospitals may make it challenging to demonstrate mortality reductions over the short term [46]. High-quality data platforms may therefore be especially helpful to track whether hospital quality of care and mortality rates are improving over the long-term. Hospital neonatal outcomes may also be influenced by the successful scaling up of key interventions such as CPAP and KMC. It is therefore essential to be able to track their adoption at scale. However, outside specific research studies these data are rarely reported and the effects of programmes supporting such scaling up therefore remain largely unknown. The CIN-N data platform this study described, by spanning aspects of care rarely included in other LMIC quality assessment approaches, offered one means to track adoption over the long-term and provides a hypothesis generating platform for implementation research linked to observed variations in quality of care and intervention rollout [42, 47, 48].

### Implications of findings

For key clinical data domains (i.e., demographic, admission, and discharge information) there was good data documentation at the time hospitals joined the CIN-N. This is likely attributable to most of these sites being already part of CIN-Paediatrics, where organisationally, the learning health system culture and activities explained in detail elsewhere [13–16] were already being cultivated, allowing these hospitals to take advantage of the roll-out and dissemination of tools like the NAR coupled with ETAT+ training. This also does suggest that it is possible to introduce tools that capture essential clinical data with missingness rates of 20% or less in routine SSA hospital settings. Analyses of such data do then need to account for missingness using appropriate statistical techniques to reduce potential biases [49, 50]. The slow but steady month-to-month improvement illustrates how long it takes to change clinical behaviours for some forms of patient documentation.

For several documentation domains (e.g., *Other physical examination*, *Maternal History*, *Monitoring Vital Signs*) and all treatment accuracy and coverage domains except antibiotic prescribing accuracy, considerable variability in performance between hospitals remains a persistent challenge.

Domains that had lower baselines were those where documentation practices were less standardised prior to joining CIN-N. New information tools such as Transfer Forms (for sick newborns transferred from labour wards or theatres to NBU) or feedback on documentation of vital signs on NBU admission may have improved performance for *Maternal history* and *Monitoring (Vital signs)* domains across hospitals over time (Figs 2 and 3). However, the challenges with adoption or improvement were illustrated by both patterns where facilities starting very low showed gradual improvement and those starting high which either stagnated or got worse.

In some cases, this may reflect a “ceiling” effect (e.g., *Fluids* prescribing accuracy). It is evident, however, that some hospitals could attain higher accuracy levels consistently (Figs 3 and 5), suggesting improvements in other sites would be possible. Similarly, trends in accuracy in the prescription of feeds and fluids are quiet erratic and vary within and between hospitals (Fig 5). These erratic patterns might have been exacerbated by the limited interaction of hospitals through CIN meetings during the COVID-19 pandemic and other challenges to sustaining quality care such as human resource shortages and labour strikes [19, 20]. Learning from ‘positive deviants’ may be informative. Prior research suggests good performers have adequate and supportive staffing, participate actively in local clinical audits and feedback process, and have good supervision by unit leads [14–16, 51].

Better theory-driven ways of conducting audit and feedback might be required within CIN-N to improve quality of care and treatment accuracy [33]. For example, more active feedback might be needed for more complex tasks such as to promote accurate prescribing. Further elaborations might explicitly address (1) capacity limitations of CIN-N hospitals and clinical teams to produce the improvements required, (2) lessons learned about the identity and culture of each individual CIN-N hospital and site specific barriers to change (3) specific use of behavioural thinking that directly supports positive clinical behaviours by ensuring feedback is actionable, controllable and timely [40, 52].

### Strengths and limitations

This study is among the few in SSA focusing on documentation of new-born data, an implementation of strategic objective 2 and 5 of the Every Newborn Action Plan [6, 53, 54]. The CIN-N generates data from more than 20 NBUs across Kenya by improving routine data sources in a strategy that is relatively low-cost and scalable as the central data management and data quality assurance processes involved can benefit from economies of scale [9, 55]. However, the data generated are limited only to ‘documentation’, limiting the range of measures of quality e.g., whether prescriptions were correct. Key limitations therefore remain such as confirmation of whether treatment was dispensed as prescribed (i.e., treatment adherence), and, for interventions such as CPAP, it can be hard to determine the best denominator population which would ideally be newborns that might have benefited from its use. This problem in identifying suitable denominators that enable evaluation of the appropriate use of interventions mean tracking adoption may frequently be based on a cruder measure of frequency of (documented) use. Furthermore, for some indicators applicable to only relatively small numbers of patients performance may appear erratic because there are few data points per month. Thus, less frequent monitoring over longer time periods may be required to sensibly track trends.

## Conclusions

All neonatal in-patient care documentation domains demonstrated modest improvements between 0.6% and 7.6% per month in the number of patients with complete domain documentation with each month in the CIN. From the improved clinical data quality, evaluation of essential treatment accuracy was possible, with the data showing a month-to-month improvement in prescribing accuracy of 2.8% and 1.4% for feeds and fluids but not for Antibiotic prescriptions after hospitals joining the CIN.

It is possible to introduce tools that capture essential clinical data often in 80% or more of newborns admitted to routine SSA hospital settings engaged in a centrally supported peer-to-peer network, but analyses of such data need to account for missingness using appropriate statistical techniques. Improvements in quality indicators are on average modest but valuable on a month-to-month basis and occur over a prolonged period that included the COVID pandemic. Average effects mask considerable temporal and between hospital variability with some hospitals demonstrating high levels of performance for indicators likely to be important to patient safety and outcomes such as feeding or antibiotic treatment prescribing accuracy. Future research is needed to explore how learning from high-performing hospitals in a learning health system in SSA context can help realise better improvements more widely within the hospital network while continuously deploying better theory-driven feedback approaches. However, considerable system challenges such as rapid staff turnover, general staff shortages and ongoing material resource challenges likely contribute to persistent problems delivering quality care. Such quality clinical data (and associated platforms) can support better impact evaluation, performance benchmarking, exploration of links between health system inputs and outcomes and critical scrutiny of geographic variation in quality and outcomes of hospital care [56]. Efforts to improve the quality of clinical data from SSA are needed to support these objectives remain much needed.

## Supporting information

S1 Checklist STROBE checklist. (DOCX)

S1 Table Hospitals’ CIN-N membership and patient volumes. (DOCX)

S2 Table Sensitivity analysis evaluating if there is any evidence that negative binomial model assumptions might have been violated. (DOCX)

S1 Fig Hospital-specific documentation trends for last half of randomly selected CIN-N hospitals. Trend line generated using LOWESS technique. Fewer observations in some hospitals due to different CIN-N joining dates.
(TIF)

S2 Fig Hospital-specific treatment accuracy trends for half of randomly selected CIN-N hospitals. Trend line generated using LOWESS technique. Fewer observations in some hospitals due to different CIN-N joining dates.
(TIF)

S3 Fig Normality assumption check for Poisson and negative binomial generalised linear mixed effects models for documentation completeness domains. (TIF)

S4 Fig Linearity assumption check for Poisson and negative binomial generalised linear mixed effects models the documentation completeness domains. (TIF)

S5 Fig Linearity and normality assumption check for the treatment appropriateness Poisson and negative binomial generalised linear mixed effects models. (TIF)

S1 Acknowledgments (DOCX)

## Abbreviations

A&F: Audit and Feedback
CIN: Clinical Information Network
CPAP: Continuous Positive Airway Pressure
ETAT+: Emergency Treatment and Triage plus admission
KEMRI: Kenya Medical Research Institute
KMC: Kangaroo Mother Care
KPA: Kenya Paediatric Association
LMICs: Low and middle-income countries
MoH: Ministry of Health; NAR, Neonatal Admission Record
NBU: Newborn Unit; SERU, KEMRI’s Scientific and Ethics Review Unit
SSA: Sub-Saharan Africa; WHO, World Health Organization
