# Supplementary material for: Improving in-patient neonatal data quality as a pre-requisite for monitoring and improving quality of care at scale: A multisite retrospective cohort study in Kenya
Source: PLOS Glob Public Health. 2022 Oct 20;2(10):e0000673. doi: 10.1371/journal.pgph.0000673 (PMC10021237; doi:10.1371/journal.pgph.0000673)
Supplement: S1 Table — (DOCX) [file pgph.0000673.s002.docx]

| S1 Table: Hospitals' CIN-N membership and patient volumes | | | | |
| --- | --- | --- | --- | --- |
| Hospital | **CIN-N Join Date^1^** | **Total Patients^2^** | **Months^3^** | **Median monthly patient count (Inter-quartile range)** |
| H1 | November 2018 | 3815 | 36 | 106 (96-117) |
| H2 | February 2018 | 2628 | 45 | 58 (50-64) |
| H3 | March 2018 | 5245 | 44 | 116 (100-144) |
| H4 | June 2019 | 683 | 28 | 23 (22-29) |
| H5 | November 2018 | 3142 | 36 | 90 (73-107) |
| H6 | April 2018 | 5544 | 43 | 130 (121-137) |
| H7 | March 2018 | 4408 | 43 | 98 (92-106) |
| H8 | October 2018 | 7690 | 37 | 217 (201-232) |
| H9 | March 2018 | 1785 | 43 | 45 (29-52) |
| H10 | April 2018 | 3787 | 35 | 85 (61-100) |
| H11 | March 2018 | 8836 | 44 | 198 (178-209) |
| H12 | June 2017 | 8606 | 46 | 185 (158-208) |
| H13 | March 2018 | 3486 | 44 | 76 (60-94) |
| H14 | November 2019 | 2160 | 24 | 92 (78-99) |
| H15 | September 2019 | 3550 | 18 | 139 (129-147) |
| H16 | September 2019 | 5334 | 26 | 188 (175-201) |
| H17 | April 2018 | 4264 | 43 | 95 (72-106) |
| H18 | October 2018 | 7311 | 37 | 200 (169-242) |
| H19 | March 2018 | 1533 | 44 | 34 (28-43) |
| H20 | July 2017 | 1153 | 40 | 23 (18-29) |
| Note:  ^1^Hospitals joined at different calendar times.  ^2^Monthly patient counts includes all data before any exclusion.  ^3^Data collected between January 2017 and December 2017, and December 2020 and January 2021 omitted due to a national health workers’ strike (i.e., industrial action) | | | | |
