# Supplementary material for: Improving in-patient neonatal data quality as a pre-requisite for monitoring and improving quality of care at scale: A multisite retrospective cohort study in Kenya
Source: PLOS Glob Public Health. 2022 Oct 20;2(10):e0000673. doi: 10.1371/journal.pgph.0000673 (PMC10021237; doi:10.1371/journal.pgph.0000673)
Supplement: S2 Table — (DOCX) [file pgph.0000673.s003.docx]

| S2 Table: Sensitivity analysis evaluating if there is any evidence that negative binomial model assumptions might have been violated? | | | | | | | | |
| --- | --- | --- | --- | --- | --- | --- | --- | --- |
| Domain^1^ | **Over-dispersion value^2^** | **Equi-dispersion**  **LRT p-value^2^** | **Nested model LRT**  **p-value^3^** | **Auto-correlation LRT p-value^4^** | **AIC** | **BIC** | **Log likelihood** | **Deviance** |
| Admission Information | 0 | 1 | <0.001 | <0.001 | 4988.35 | 5015.89 | -2488.18 | 4976.35 |
| Cardinal Signs | 0.05 | 0.581 | <0.001 | <0.001 | 6071.43 | 6099.19 | -3029.71 | 6059.43 |
| Demographics | 0 | 0.915 | <0.001 | <0.001 | 5187.99 | 5215.76 | -2588 | 5175.99 |
| Discharge Information | 0.3 | 0.03 | <0.001 | <0.001 | 6363.95 | 6391.72 | -3175.97 | 6351.95 |
| Maternal History | 0.117 | 0.303 | <0.001 | <0.001 | 6012.4 | 6040.17 | -3000.2 | 6000.4 |
| Monitoring (Vital Signs) | 2.254 | <0.001 | <0.001 | <0.001 | 5124.04 | 5151.66 | -2556.02 | 5112.04 |
| Other Examinations | 2.442 | <0.001 | <0.001 | <0.001 | 6176.34 | 6204.11 | -3082.17 | 6164.34 |
| Presenting Complaints | 0 | 0.902 | <0.001 | <0.001 | 6291.42 | 6319.18 | -3139.71 | 6279.42 |
| Antibiotics | 0 | 0.945 | 0.097 | <0.001 | 4477.24 | 4504.85 | -2232.62 | 4465.24 |
| Feeds^4^ | 0.571 | 0.001 | <0.001 | <0.001 | 2263.05 | 2289.49 | -1125.52 | 2251.05 |
| Fluids | 0 | 1 | <0.001 | <0.001 | 4171.96 | 4199.22 | -2079.98 | 4159.96 |
| Note:  ^1^Evidence from all the final documentation completeness and treatment appropriateness negative binomial and Poisson models fitted (Tables 5 and 6) is suggestive that the model errors are normally distributed (i.e., the conditional outcomes were normally distributed) and log-linear (S3 Fig, S4 Fig, S5 Fig in the supporting information) thus suitable for these analyses.  ^2^Model comparison test between negative binomial- and Poisson model structure for evidence in supporting presence of overdispersion.  ^3^Comparison test between random slope-intercept model and random intercept only models for evidence in support of allowing time to vary within hospitals  ^4^Comparison between random slope-intercept model (for time and hospital respectively) and random intercept model with auto-correlation term for time for exploring how best to allow time to vary within hospitals during model specification. | | | | | | | | |
