# Supplementary figures and images for: Improving in-patient neonatal data quality as a pre-requisite for monitoring and improving quality of care at scale: A multisite retrospective cohort study in Kenya
Source: PLOS Glob Public Health. 2022 Oct 20;2(10):e0000673. doi: 10.1371/journal.pgph.0000673 (PMC10021237; doi:10.1371/journal.pgph.0000673)

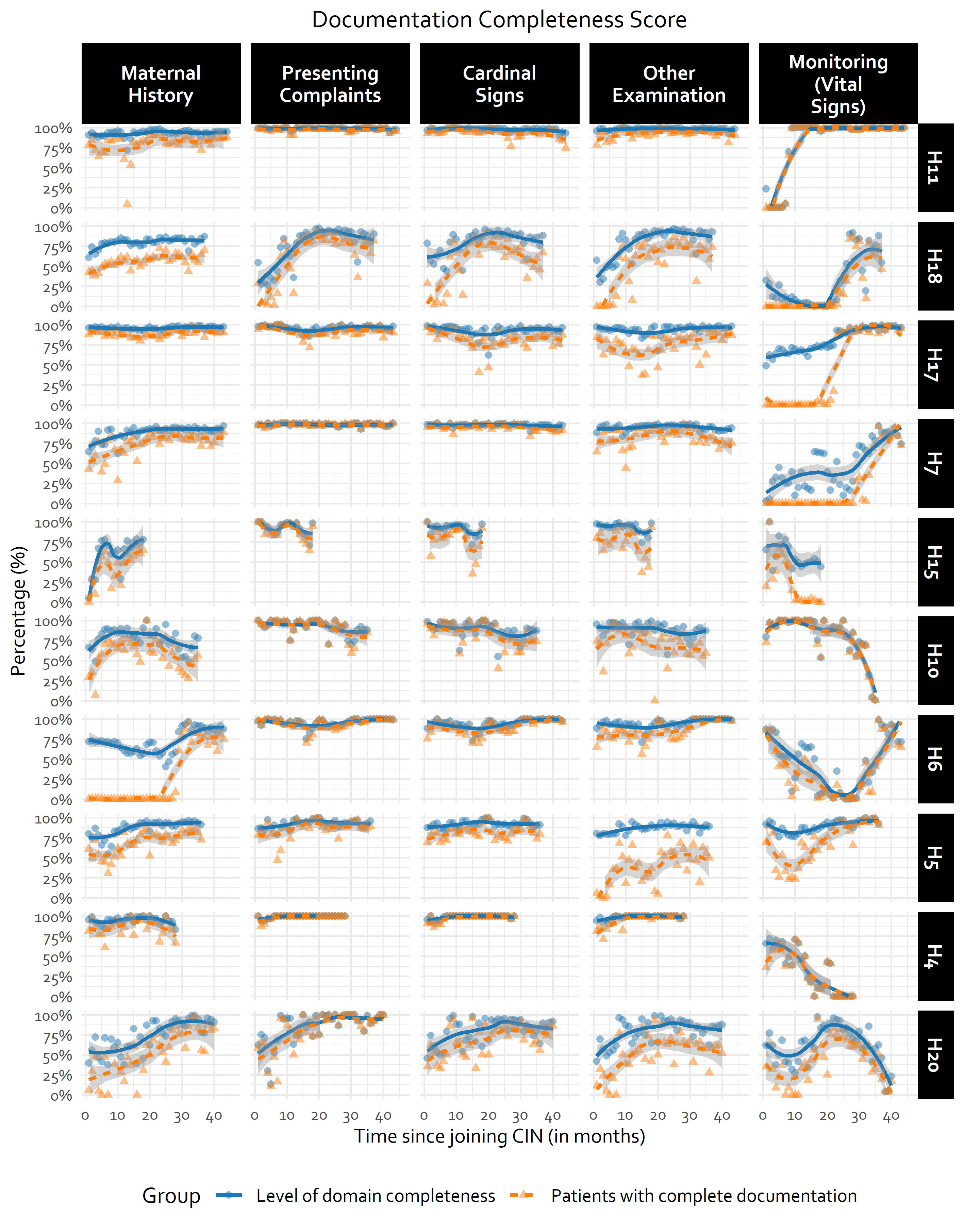

Supplement: S1 Fig — Trend line generated using LOWESS technique. Fewer observations in some hospitals due to different CIN-N joining dates. (TIF) [file pgph.0000673.s004.tif]

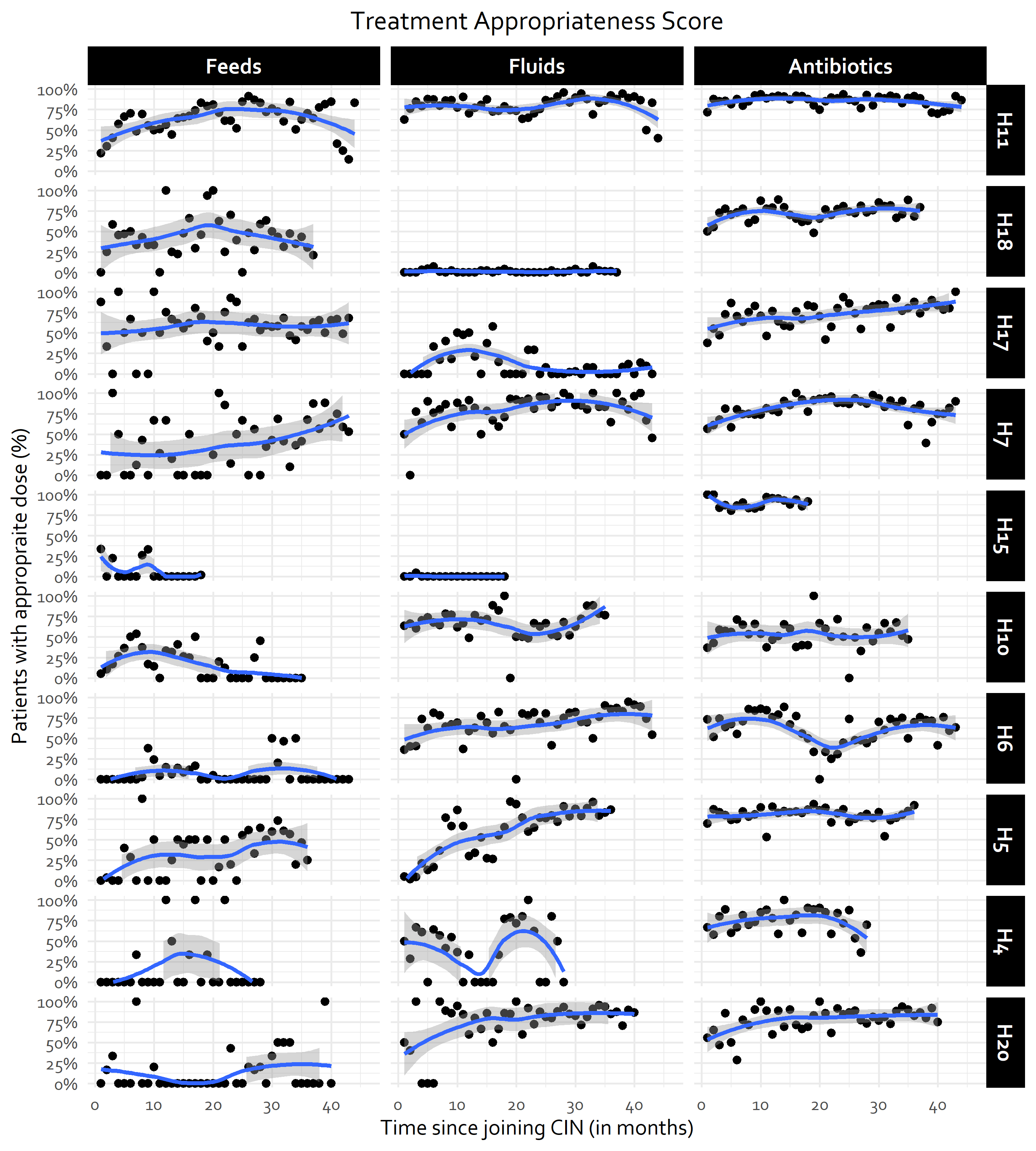

Supplement: S2 Fig — Trend line generated using LOWESS technique. Fewer observations in some hospitals due to different CIN-N joining dates. (TIF) [file pgph.0000673.s005.tif]

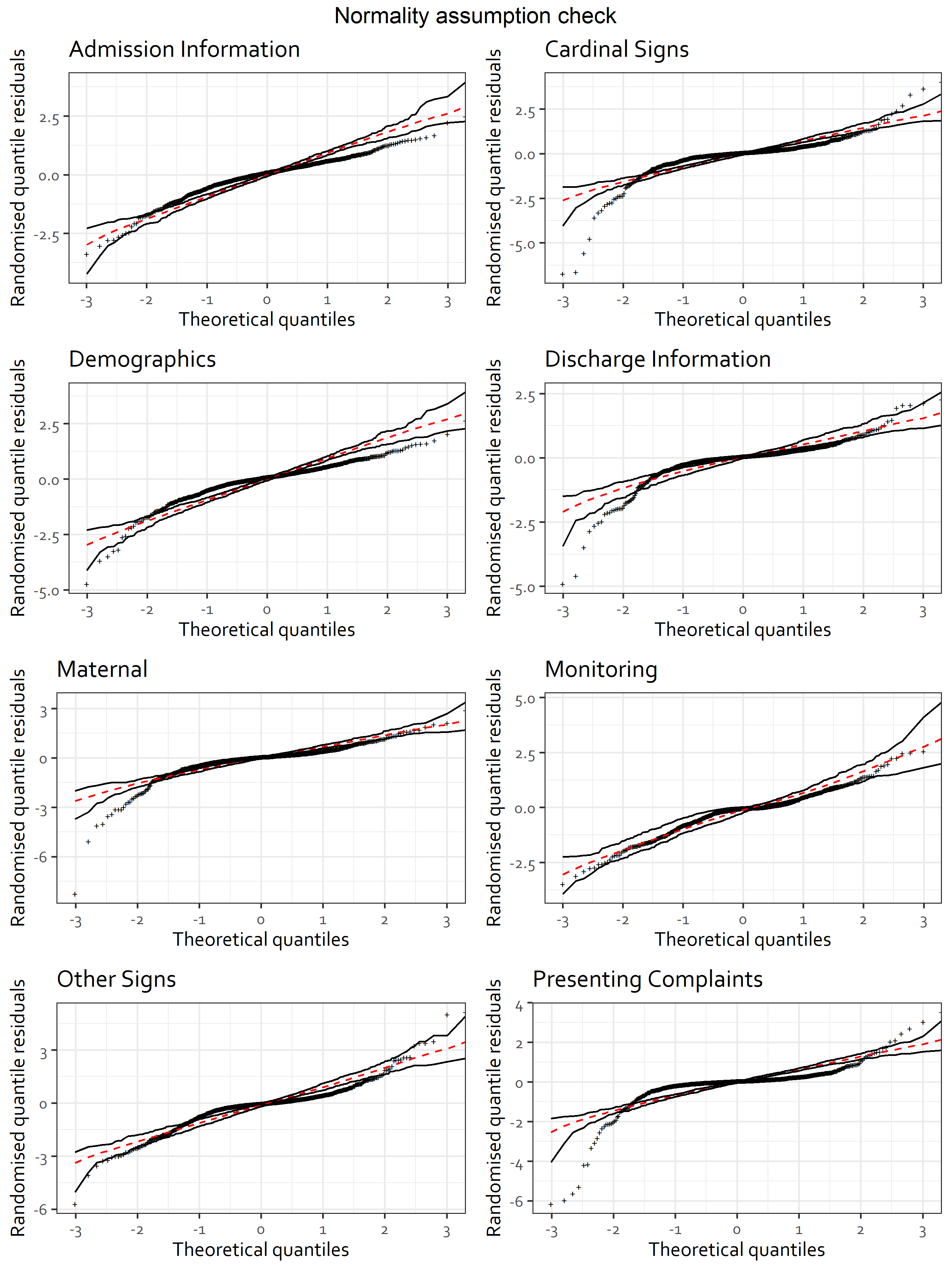

Supplement: S3 Fig — (TIF) [file pgph.0000673.s006.tif]

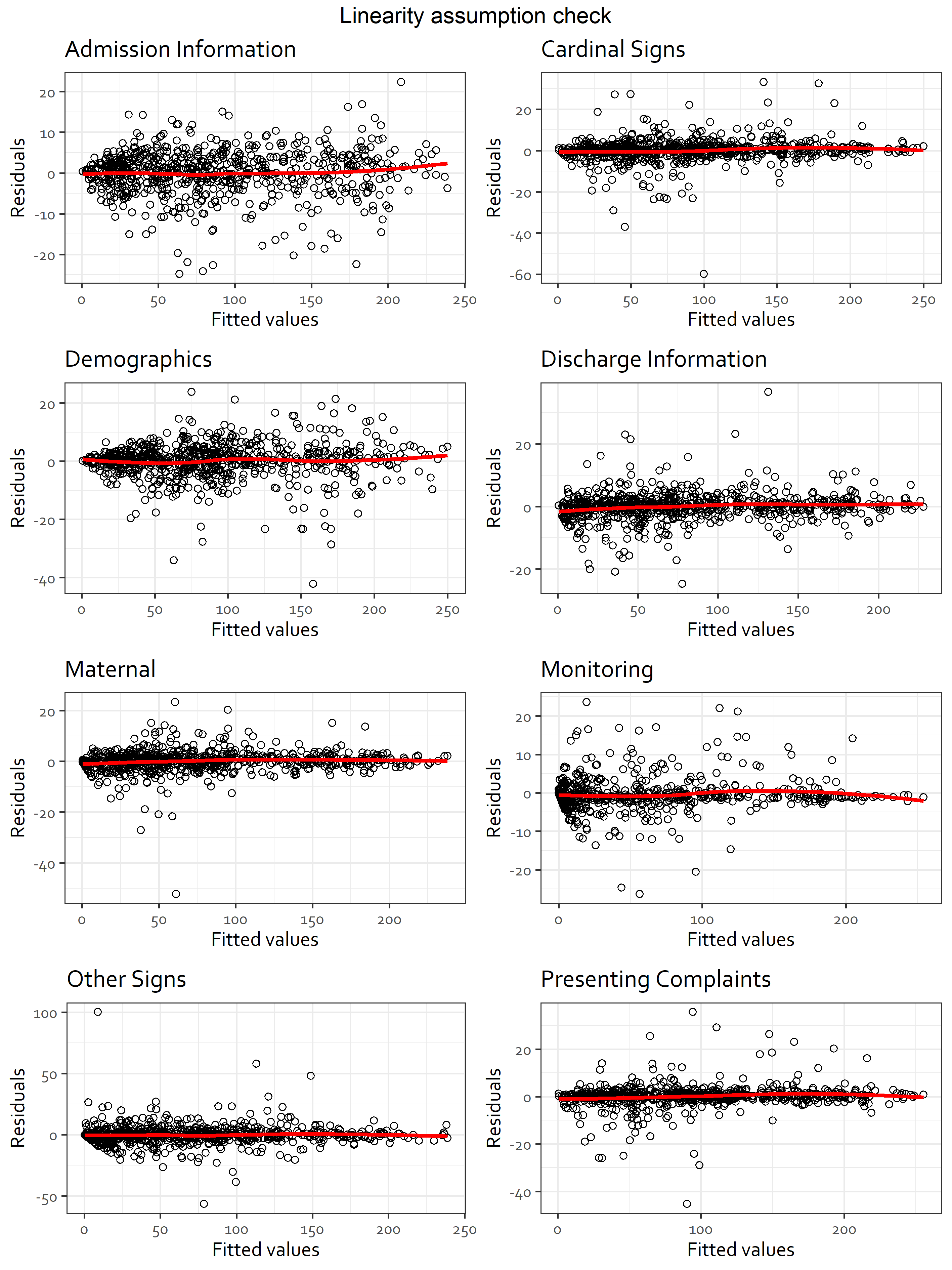

Supplement: S4 Fig — (TIF) [file pgph.0000673.s007.tif]

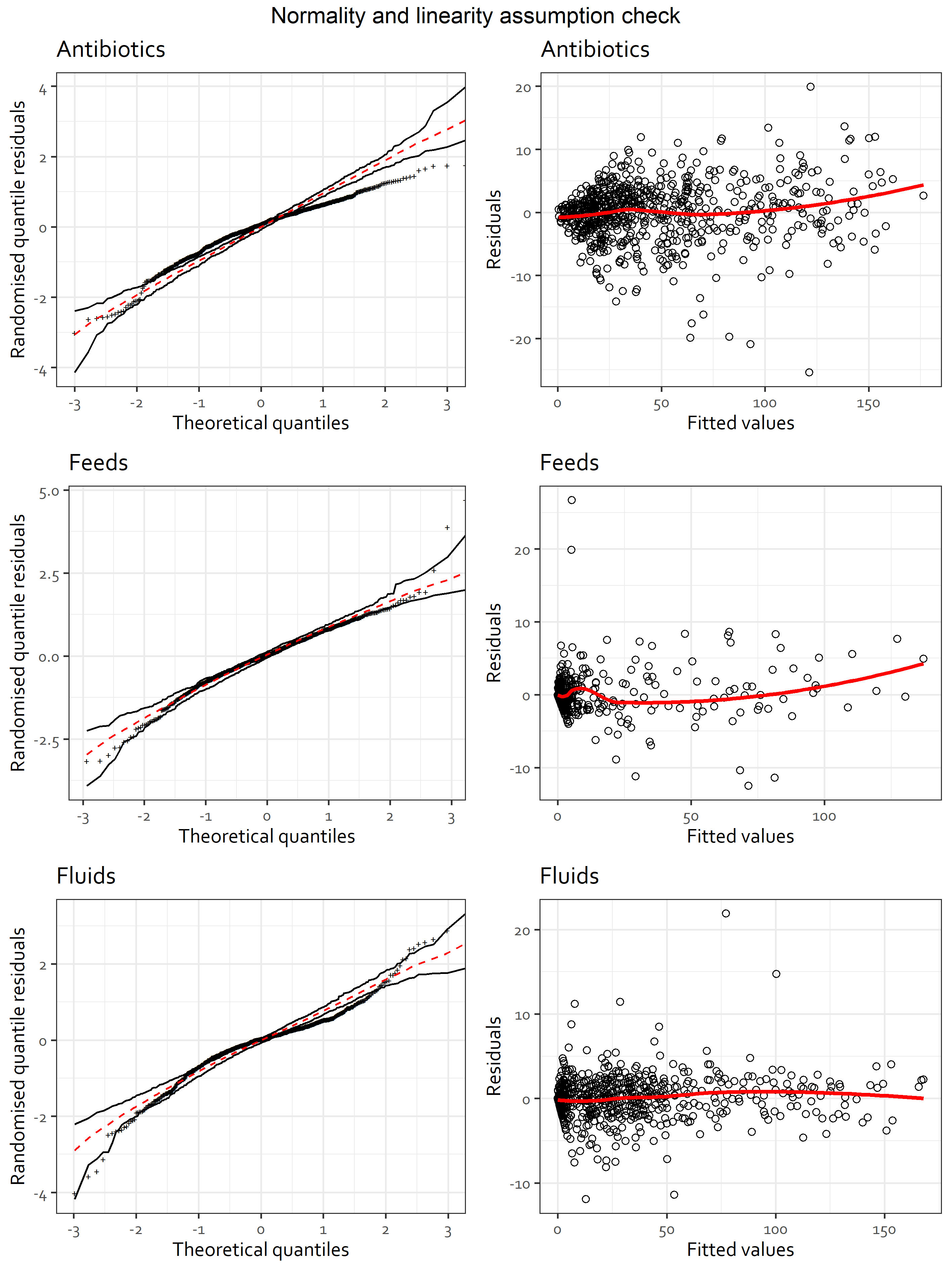

Supplement: S5 Fig — (TIF) [file pgph.0000673.s008.tif]
