# Supplementary material for: Improving in-patient neonatal data quality as a pre-requisite for monitoring and improving quality of care at scale: A multisite retrospective cohort study in Kenya
Source: PLOS Glob Public Health. 2022 Oct 20;2(10):e0000673. doi: 10.1371/journal.pgph.0000673 (PMC10021237; doi:10.1371/journal.pgph.0000673)
Supplement: S1 Acknowledgments — (DOCX) [file pgph.0000673.s009.docx]

### **Acknowledgements**

The **Clinical Information Network (CIN) Group**: The CIN group hospital teams who are tagged to collaborate in the network’s development, data collection, data management, implementation of audit and feedback interventions and who will participate in this study include the following focal persons:

1. **Paediatricians:** Juma Vitalis, Nyumbile Bonface, Roselyne Malangachi, Christine Manyasi, Catherine Mutinda, David Kibiwott Kimutai, Rukia Aden, Caren Emadau, Elizabeth Atieno Jowi, Cecilia Muithya, Charles Nzioki, Supa Tunje, Dr. Penina Musyoka, Wagura Mwangi, Agnes Mithamo, Magdalene Kuria, Esther Njiru, Mwangi Ngina, Penina Mwangi, Rachel Inginia, Melab Musabi, Emma Namulala, Grace Ochieng, Lydia Thuranira, Felicitas Makokha, Josephine Ojigo, Beth Maina, Catherine Mutinda, Mary Waiyego, Bernadette Lusweti, Angeline Ithondeka, Julie Barasa, Meshack Liru, Elizabeth Kibaru, Alice Nkirote Nyaribari, Joyce Akuka, Joyce Wangari;
2. **Nurses:** Amilia Ngoda, Aggrey Nzavaye Emenwa, Patricia Nafula Wesakania, George Lipesa, Jane Mbungu, Marystella Mutenyo, Joyce Mbogho, Joan Baswetty, Ann Jambi,  Josephine Aritho, Beatrice Njambi, Felisters Mucheke, Zainab Kioni, Jeniffer, Lucy Kinyua, Margaret Kethi, Alice Oguda, Salome Nashimiyu Situma, Nancy Gachaja, Loise N. Mwangi, Ruth Mwai, irginia Wangari Muruga, Nancy Mburu, Celestine Muteshi, Abigael Bwire, Salome Okisa Muyale, Naomi Situma, Faith Mueni, Hellen Mwaura, Rosemary Mututa, Caroline Lavu, Joyce Oketch, Jane Hore Olum, Orina Nyakina, Faith Njeru, Rebecca Chelimo, Margaret Wanjiku Mwaura, Ann Wambugu, Epharus Njeri Mburu, Linda Awino Tindi, Jane Akumu, Ruth Otieno, Slessor Osok;
3. **Health Record Information Officers (HRIOs):** Seline Kulubi, Susan Wanjala, Pauline Njeru, Rebbecca Mukami Mbogo, John Ollongo, Samuel Soita, Judith Mirenja, Mary Nguri, Margaret Waweru, Mary Akoth Oruko, Jeska Kuya, Caroline Muthuri, Esther Muthiani, Esther Mwangi, Joseph Nganga, Benjamin Tanui, Alfred Wanjau, Judith Onsongo, Peter Muigai, Arnest Namayi, Elizabeth Kosiom, Dorcas Cherop, Faith Marete, Johanness Simiyu, Collince Danga, Arthur Otieno Oyugi, Fredrick Keya Okoth.

The **Clinical Information Network (CIN) Group**’s monitored email address is [CIN@kemri-wellcome.org](mailto:CIN@kemri-wellcome.org) and the list can change when new paediatrician(s), nurse(s) or HRIO leave or come into the hospital.
